# Reproductive Phase Locking of Mosquito Populations in Response to Rainfall Frequency

**DOI:** 10.1371/journal.pone.0000331

**Published:** 2007-03-28

**Authors:** Jeffrey Shaman, Jonathan F. Day

**Affiliations:** 1 College of Oceanic and Atmospheric Sciences, Oregon State University, Corvallis, Oregon, United States of America; 2 Florida Medical Entomology Laboratory, Institute of Food and Agricultural Sciences, University of Florida, Vero Beach, Florida, United States of America; University of Sydney, Australia

## Abstract

The frequency of moderate to heavy rainfall events is projected to change in response to global warming. Here we show that these hydrologic changes may have a profound effect on mosquito population dynamics and rates of mosquito-borne disease transmission. We develop a simple model, which treats the mosquito reproductive cycle as a phase oscillator that responds to rainfall frequency forcing. This model reproduces observed mosquito population dynamics and indicates that mosquito-borne disease transmission can be sensitive to rainfall frequency. These findings indicate that changes to the hydrologic cycle, in particular the frequency of moderate to heavy rainfall events, could have a profound effect on the transmission rates of some mosquito-borne diseases.

## Introduction

Mosquito populations and the transmission of mosquito-borne pathogens are sensitive to both temperature and hydrologic variability [1]. As the climate changes in response to increasing atmospheric greenhouse gas concentrations [2], higher temperatures and changes to the hydrologic cycle are expected to affect both mosquito population dynamics and transmission rates of mosquito-borne diseases. Much debate has been given to the possible effects of increasing temperatures on vector-borne disease transmission patterns [3]–[5]; however, future hydrologic changes are likely to be more important determinants of transmission rates than global warming.

Researchers have long looked for associations between rainfall variability and mosquito abundance [6]–[9] and mosquito-borne disease incidence [10]–[16]. While it is convenient to use rainfall amount as an explanatory hydrologic variable, the physical effects of precipitation on environmental conditions are multiple, and the responses of different mosquito species and mosquito-borne pathogens to these effects are varied. As a result, it is often difficult to establish significant and stationary relationships between the amount of precipitation and mosquito abundance or mosquito-borne disease transmission patterns.

Rainfall has two principal influences on the mosquito life cycle: 1) the increased near-surface humidity associated with rainfall enhances mosquito flight activity and host-seeking behavior, and 2) rainfall can alter the abundance and type of aquatic habitats available to the mosquito for the deposition of eggs (oviposition) and the subsequent development of the immature stages. In this study we focus primarily on the first influence. Moderate to heavy rainfall can synchronize mosquito population activity by increasing near-surface humidity levels and stimulating resting gravid mosquitoes to oviposit and then seek out new hosts [17]–[23]. A portion of these mosquitoes may be infectious (e.g. with West Nile virus) and will transmit these infectious agents to any susceptible hosts upon which they feed. Thus, moderate to heavy rainfall events can stimulate episodes of disease transmission, and repeated rainfalls may increase disease transmission to epidemic levels. This biology suggests that the frequency of rainfall events may be an important determinant of mosquito population dynamics and rates of mosquito-borne transmission.

Here we explore this effect of rainfall event frequency on mosquito population dynamics. We present a simple model that characterizes the mosquito reproductive cycle as a forced phase oscillator. The forcing term represents rainfall, which stimulates the host-seeking, blood-feeding, and egg-laying behaviors of female mosquitoes and, in so doing, synchronizes the age and reproductive structure of the model mosquito population.

## Results

The mosquito reproductive cycle is modeled as:1
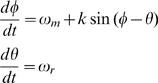
where *Φ* is the phase of the mosquito reproductive cycle, *θ* is the phase of the rainfall cycle, *ω_m_* is the natural frequency of mosquito reproductive cycle, *ω_r_* is the frequency of rainfall events, and *k* is the coupling coefficient. The cycles are defined such that when *Φ* equals 0, parous female mosquitoes oviposit and then blood feed; when *θ* equals 0, rainfall occurs. The natural frequency, *ω_m_*, is the rate at which the eggs within the female mosquito develop during an average reproductive cycle from blood feeding to oviposition, assuming optimal temperature and rainfall conditions. In form, Equation 1 resembles a coupled oscillator in which the phase of the mosquito reproductive cycle has no influence on rainfall phase.

**Figure 1:**
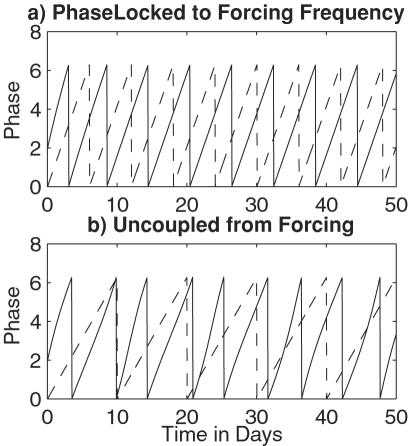
Plots of phase versus time from Equation 1. Solid lines are the phase of the mosquito reproductive cycle; dashed lines are the rainfall phase. a) System in which the mosquito reproductive cycle resonates at the rainfall phase forcing; *ω_m_* = 2π/(5 days); *ω_r_ = *2π/(6 days); *k* = 0.35/day. b) System in which the mosquito reproductive cycle is uncoupled from the rainfall phase forcing; *ω_m_* = 2π/(5 days); *ω_r_ = *2π/(10 days); *k* = 0.35/day. Phase values range from 0 to 2π.

Phase oscillators such as Equation 1 have some well-known dynamical properties [24]. Specifically, if *|ω_m_−ω_r_|<k* then the mosquito reproductive cycle will phase-lock or resonate at the forcing frequency *ω_r_*. If *|ω_m_−ω_r_|>k* then the mosquito reproductive cycle will uncouple from the forcing frequency (Figure 1). Female mosquitoes blood-feed to provide protein for egg development. Once the eggs have developed and are ready to be oviposited, some mosquito species will wait for a moderate or heavy rainfall that increases near surface humidity, producing conditions conducive for flight, oviposition, and another round of host-seeking [17]–[23]. The forced resonance that occurs when *|ω_m_−ω_r_|<k* represents this biological response to rainfall. The uncoupled phase oscillations that occur when *|ω_m_−ω_r_|>k* are biologically unrealistic (i.e. the mosquitoes no longer respond to the rainfall stimulus) and will not be explored here; however, the point at which the system uncouples (i.e. *|ω_m_−ω_r_| = k)* provides a means for estimating the coupling coefficient (see Materials and Methods).

This phase oscillator (Equation 1) is coupled to a very simple model of mosquito biology (see Materials and Methods). The model assumes optimal temperature conditions and constant mortality and birth rates. Two mosquito population classes are simulated: 1) parous females (those that have blood fed and oviposited at least once and are thus potentially infected with a mosquito-borne pathogen) and 2) cohorts of mosquitoes at all other stages of life—egg, larvae, pupae, and nulliparous (those that have never blood fed) adult females. Parous and nulliparous mosquitoes respond to rainfall events by attempting to complete a reproductive cycle (host seeking, blood feeding, egg maturation, and oviposition). This reproductive response is determined by Equation 1.

Our parameter choice endeavors to simulate Culex nigripalpus biology. In Florida, this mosquito species is the proven primary vector of St. Louis encephalitis virus (SLEV) [25]–[27] and the suspected primary vector of West Nile virus (WNV) [28]–[29]. Culex nigripalpus prefers newly flooded fresh water breeding habitats that contain recent organic infusions, especially those habitats laden with dried grass. Prime oviposition habitats for this species include freshly flooded agricultural, residential, and urban irrigation and runoff furrows where standing water develops after a heavy rain, persists for approximately 10 days and then dries up. This dry-down allows a reconstitution of the infusion when the area re-floods. Under ideal conditions, habitats that are re-flooded every 10 days are preferred oviposition sites for gravid Cx. nigripalpus females. Under these conditions it takes about 6 days for development from egg to a newly emerged adult. By day 7–8 the oviposition habitat has dried down and by day 10 the vegetation and soil is completely dry and ready for re-flooding to produce a new infusion and a suitable oviposition habitat [30]–[31]. Culex nigripalpus females will retain their eggs for weeks or even months waiting for the creation of suitable flight conditions and oviposition sites by heavy (>50 mm) rainfall events [22]. This biology suggests that the natural period, or ‘ideal reproductive cycle,’ for Cx. nigripalpus females is approximately 10 days (ω_m_ = 2π/(10 days)). We use 40 days as the period of decoupling in our estimation of the coupling coefficient.

**Figure 2:**
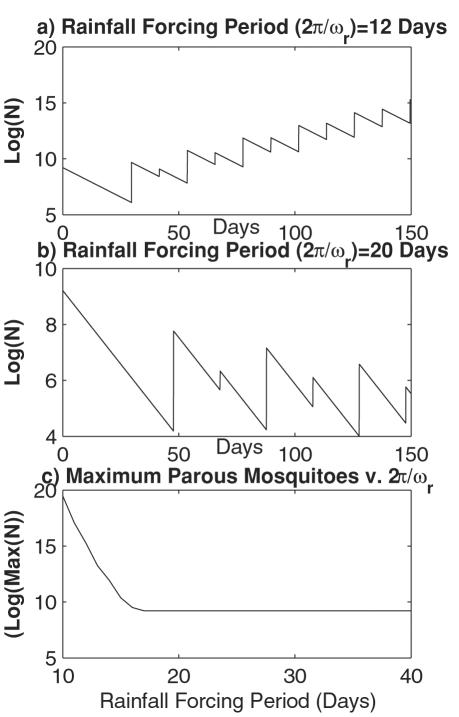
Simulation of *Cx. nigripalpus* response to rainfall events of fixed frequency. a) Log plot of the simulated *Cx. nigripalpus* parous population (*N*). The initial population is *N* = 10,000 at *t* = 0. *ω_m_* = 2π/(10 days); *ω_r_* = 2π/12 days; *k* = 0.47/days, or uncoupling ∼40 days; *r* = 35/hour; *d* = 0.0044/hour or 10% mortality per day. b) Log plot of the simulated *Cx. nigripalpus* parous population (*N).* The initial population is *N* = 10,000 at *t* = 0. *ω_m_* = 2π/(10 days); *ω_r_* = 2π/20 days; *k* = 0.47/days, *r* = 35/hour; *d* = 0.0044/hour. c) Log plot of the maximum number of parous mosquitoes over the course of 150-day runs, plotted as a function of rainfall forcing period (2π/*ω_r_*). The initial population of each run is *N* = 10,000 at *t* = 0. *ω_m_* = 2π/(10 days); *k* = 0.47/days; *r* = 35/hour; *d* = 0.0044/hour.

Figure 2a shows that with a rainfall frequency (ω_r_) near the frequency of the ideal oviposition cycle (ω_m_), the model Cx. nigripalpus population increases. However, as this rainfall frequency decreases the phase-locked population responds with less frequent reproduction and dwindling numbers (Figure 2b). Figure 2c shows how a population of parous Cx. nigripalpus females cycles over the course of 150-day run and how these population cycles are influenced by rainfall period. A critical point lies at the 16-day period. The Cx. nigripalpus population increases in response to rainfall at frequencies higher than this critical point. Rainfall forcing at lower frequencies produces insufficient reproduction to maintain the Cx. nigripalpus population; instead, the maximum number of mosquitoes is the initial number and the population declines as in Figure 2b.

**Figure 3:**
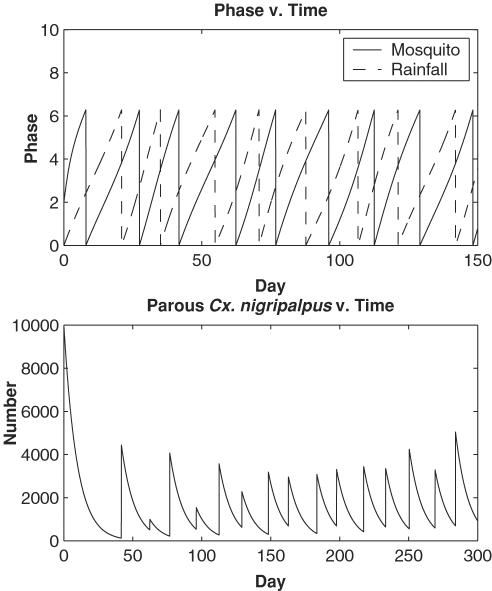
Simulation of *Cx. nigripalpus* response to rainfall events of varying frequency. a) Plot of mosquito (Φ) and rainfall (θ) phase over the first 150 days of a 300-day simulation. b) Plot of the parous *Cx. nigripalpus* population over the entire run. The initial population is *N* = 10,000 at *t* = 0. *ω_m_* = 2π/(10 days); *ω_r_* = 2π/(18+5*sin(2πt/40) days); *k* = 0.47/days, or uncoupling ∼40 days; *r* = 35/hour; *d* = 0.0044/hour or 10% mortality per day. The time-dependent sine function in the denominator of the rainfall frequency term (*ω_r_*) produces a stable mosquito population.

By making ω_r_ a function of time that oscillates about the 16-day critical period, a stable population of mosquitoes is realized (Figure 3). That is, by simply varying the frequency of rainfall to which the ideal oviposition cycle resonates, Cx. nigripalpus birth and death rates approximately match over the course of the model simulation. In the example presented, ω_r_ includes a time-dependent sinusoidal term, such that the time between rainfall events ranges between 13 and 23 days.

**Figure 4:**
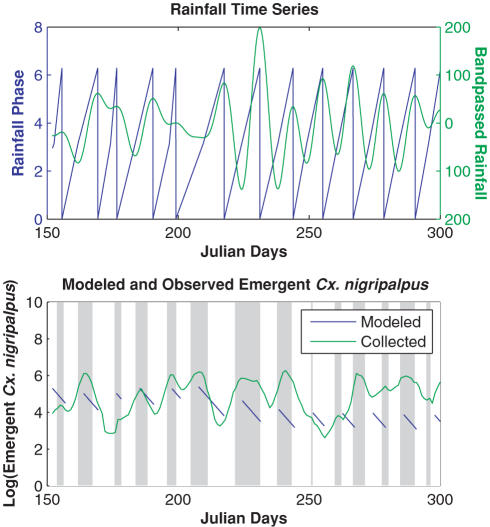
Full model simulation of emergent *Cx. nigripalpus* population response to measured rainfall frequency. a) Time series of 10–40 period bandpassed rainfall and the phase of this rainfall. Raw rainfall data is from National Climate Data Center records, station Vero Beach 4SE. b) Log transform of both modeled and collected emergent *Cx. nigirpalpus.* A 5-day moving average has been applied to the collected data. Vertical bars highlight local maxima in the observed record. The simulated emergent time series is discontinuous due to model construct. The emergent population appears when cohorts finish pupation and vanishes as these mosquitoes take their first blood meal and begin egg development. The model was run for 365 days. The initial simulated population is *N* = 100 at *t* = 0 (January 1, 1990), *ω_m_* = 2π/(10 days); *k* = 0.47/days; *r* = 35/hour; *d* = 0.0068/hour or 15% mortality per day. Both plots are shown for Julian days 151–300 of 1990 (∼June–October) during which a St. Louis encephalitis epidemic occurred in Florida. Vero Beach was the epicenter of this epidemic.

We next use the model to simulate the emergence of Cx. nigripalpus during the 1990 SLEV epidemic in Indian River County, Florida. Previous work has shown that the emergence of Cx. nigripalpus during the 1990 epidemic was cross-correlated with heavy rainfall events [30]. These rainfall events took place in the late spring and early summer when both the wild bird and Cx. nigripalpus populations were already highly infectious with SLEV due to earlier springtime drought-induced amplification [32]. We used measured rainfall data from Vero Beach, Indian River County to develop a time series of rainfall phase (θ). This time series of phase values (Figure 4a) was then used to force the ideal Cx. nigripalpus reproductive cycle represented in Equation 1. Model simulations were compared with daily collections of resting emergent Cx. nigripalpus taken near Vero Beach (Figure 4b).

The timing of the appearance of the simulated emergent population matches most of the peaks in the collected emergent Cx. nigripalpus record from the late spring through summer and early fall, when temperatures were optimal for rapid mosquito development and during which heavy SLEV transmission took place (Figure 4b). This correspondence is surprisingly good given the simplicity of the model. The breakdown of this correspondence occurs in the fall (around Julian day 265, mid to late September) at which point temperatures and photoperiods had dropped and slowed mosquito development and reproductive rates. The model demonstrates that rainfall event frequency can affect mosquito population dynamics, and it corroborates previous findings associating rainfall events cycles with Cx. nigripalpus activity and the epidemic transmission of SLEV in south Florida during 1990 [30]. These model findings indicate that rainfall event frequency needs to be considered in future projections of arbovirus transmission in Florida and that rainfall event frequency might affect the population dynamics of other mosquito species and mosquito-borne disease transmission systems.

The size of the simulated emergent Cx. nigripalpus population was calibrated to match the observed population by adjusting the mortality rate and the initial population size (Figure 4b). However, the model does not capture the fluctuations in the observed population size, but instead only simulates the timing of population increases. This limitation was expected, given the model framework, which possesses greatly simplified biology, imposes a constant mortality rate, and takes no account of environmental variability (e.g. temperature, breeding habitat availability) other than rainfall event frequency. In the future, we will introduce the rainfall-phase forcing described here into a more realistic individual-based model of mosquito population dynamics that incorporates more realistic mosquito biology, variable mortality that is dependent on mosquito activity, and the effects of temperature and breeding habitat availability.

## Discussion

Previous theoretical and modeling studies have explored the effects of oscillating or varying environments on insect populations; however, theses studies have generally examined the effects of variable birth rates, mortality rates or carrying capacity on population size [33]–[35], but not the life cycle phase response of a population to environmental forcing. The model presented here indicates that such life cycle phase response is a potentially important phenomenon. Rainfall events spaced near the natural frequency of the ideal mosquito reproductive cycle produce quick phase locking and resonance, allowing the mosquito population to grow at its most efficient and exponential. Rainfall events spaced further apart than the natural frequency of the ideal mosquito reproductive cycle limit mosquito biting activity and the growth of the population to suboptimum levels.

These model findings have bearing on real-world disease systems. In previous work, we have demonstrated that a specific sequence of hydrologic conditions, spring drought followed by continued summer rainfall, is critical for the amplification and transmission of both SLEV and WNV in south Florida [32], [36]–[39]. Amplification involves a cascade of enzootic virus transmission between vector mosquitoes and wild avian hosts. Explosive amplification results in a rapid increase in the number of infected and infective vector mosquitoes. In south Florida, spring drought enhances amplification by restricting *Cx. nigripalpus* activity to select habitats, specifically densely vegetated hammocks where mosquitoes rest and wild birds nest and roost. Increased contact of *Cx. nigripalpus* and avian amplification hosts in these hammocks facilitates early season viral amplification by forcing the interaction of mosquitoes and birds.

When drought-induced, early season viral amplification in the initial transmission foci is followed by summer rainfall, which increases near surface humidity levels and the broader availability of oviposition sites, infective mosquitoes are able to disperse and initiate secondary transmission foci at sites located considerable distances from the original amplification site. This dispersal and secondary amplification in urban and suburban habitats dramatically increases the number of infective *Cx. nigripalpus* in the environment and places them into greater contact with humans during the late summer months when WNV and SLEV epidemics typically occur in South Florida. In addition, the mosquito population can increase dramatically in response to the increased water resources. Because of the increased viremic rate among the wild bird population, these newly emergent mosquitoes are themselves more likely to acquire infection.

Our previous findings have demonstrated that high transmission rates of SLEV and WNV to humans are more likely to occur after a spring drought that is followed by summer rainfall [38]–[39]. In the findings presented here, we have shown that the frequency of moderate to heavy summer rainfall events during the 1990 SLE epidemic in Florida in part determined mosquito population dynamics and the transmission biting activity of infectious mosquitoes. During 1990, the frequency of moderate to heavy rainfall events (ω_r_) recurred near the ideal natural frequency of the Cx. nigripalpus reproductive cycle (ω_m_). This timing of summertime rainfall facilitated explosive, phase-locked growth of the mosquito population, frequent biting by infectious Cx. nigripalpus, and increased transmission of SLEV.

Given these findings, which link rainfall event frequency with Cx. nigripalpus reproductive activity and the transmission of SLEV, it seems plausible that other mosquito species and systems of mosquito-borne disease transmission might be similarly affected by the frequency of rainfall events. Three genera of mosquitoes, Culex, Anopheles, and Aedes, are the principal vectors of viral encephalitides (e.g. WNV), malaria, and yellow fever and dengue, respectively. Mosquito species of all three genera respond to the increased near-surface humidity associated with rainfall with host-seeking, blood-feeding, and egg-laying behaviors [19], [40]–[42]. Our results indicate that the population growth of these mosquito species, as well as rates of encephalitis, malaria, and yellow fever and dengue transmission, could be sensitive to rainfall event frequency.

An increased frequency of heavy rainfall events occurred over mid- and high latitudes during the latter half of the 20^th^ century [2]. Continued changes to the frequency of storm events are expected as the climate warms in response to atmospheric greenhouse gas concentrations [1], [43]. The model presented here indicates that such changes might modulate the rates and efficiency with which mosquito populations grow and transmit pathogens.

## Materials and Methods

### Estimation of the Coupling Coefficient

Both *ω_m_* and the length of time, *τ*, a mosquito species will wait for rainfall can be determined experimentally. *τ* provides the time scale for the upper limit rainfall frequency, *ω_r_*, at which the system uncouples, such that *k = |ω_m_−2π/τ|*.

### Model Description

The mosquito model is as follows:
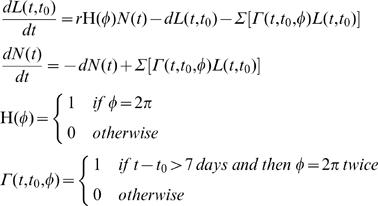
where *L(t,t_0_)* is the cohort of eggs laid at time *t = t_0_*, *r* is the birth rate, *H(Φ)* is the Heaviside function linking Equation 1 with mosquito egg-laying, *N(t)* is the number of parous female mosquitoes, *d* is the mortality rate, and Γ*(t,t_0_,Φ)* is the Heaviside function used to track cohort development from egg to parous adult. The model assumes ideal summer conditions such that initial cohort development from egg, through adult eclosion, to blood feeding readiness takes 7 days [22], [30]–[31]. At first flight following emergence (i.e. *t−t_0_>7* days and Φ = 2π), surviving mosquitoes are assumed to blood-feed; at the cohort second flight following emergence (again Φ = 2π), surviving mosquitoes are assumed to oviposit, join the parous population, *N(t)*, and blood-feed again.

### Rainfall Data

Daily rainfall data were assembled from National Climate Data Center archives for Vero Beach, Indian River County, Florida. The 1990 daily record was Fourier transformed into spectral space. A 10–40 day period bandpass was then applied and the filtered data were reverse Fourier transformed back into time space. This smoothed time series was then differentiated numerically and rainfall events were identified objectively as local maxima and minima. The local maxima and minima were then assigned phase values, θ = 0 and θ = π, respectively. The complete time series of phase values was then linearly interpolated from these values.

### Culex nigripalpus Population Data

Much of the agricultural land in Indian River County, Florida is dominated by citrus groves intermixed with hammock “islands” of southern live oak and cabbage palm [23]. Dense ground cover makes these hammocks an excellent daytime resting site for *Cx. nigripalpus* in all gonotrophic conditions (empty, parous, blood fed, and gravid) [22]. During 1990 two 20-minute aspirator collections were made daily approximately 2 hours after sunrise along a transect at a hammock site 6.4 km southwest of Vero Beach. Collected mosquitoes were sorted by species, categorized by sex and gonotrophic condition, and counted.
